# Long non-coding RNA TUG1 is up-regulated in hepatocellular carcinoma and promotes cell growth and apoptosis by epigenetically silencing of KLF2

**DOI:** 10.1186/s12943-015-0431-0

**Published:** 2015-09-04

**Authors:** Ming-De Huang, Wen-Ming Chen, Fu-Zhen Qi, Ming Sun, Tong-Peng Xu, Pei Ma, Yong-qian Shu

**Affiliations:** Department of Medical Oncology, Huai’an First People’s Hospital, Nanjing Medical University, Huai’an City, Jiangsu Province 223301 People’s Republic of China; Department of Oncology, Jining No.1 People’s Hospital, No.6, Jiankang Road, Jining City, Shandong Province 272011 People’s Republic of China; Department of Hepatopancreatobiliary Surgery, Huai’an First People’s Hospital, Nanjing Medical University, Huai’an City, Jiangsu Province 223300 People’s Republic of China; Department of Biochemistry and Molecular Biology, Nanjing Medical University, Nanjing City, Jiangsu Province People’s Republic of China; Department of Oncology, First Affiliated Hospital, Nanjing Medical University, Nanjing City, Jiangsu Province People’s Republic of China

**Keywords:** Long non-coding RNA, TUG1, HCC, Proliferation, KLF2

## Abstract

**Background:**

Hepatocellular carcinoma (HCC) is one of the leading causes of cancer-related death worldwide, and the biology of this cancer remains poorly understood. Recent evidence indicates that long non-coding RNAs (lncRNAs) are found to be dysregulated in a variety of cancers, including HCC. Taurine Up-regulated Gene 1 (TUG1), a 7.1-kb lncRNA, recruiting and binding to polycomb repressive complex 2 (PRC2), is found to be disregulated in non-small cell lung carcinoma (NSCLC) and esophageal squamous cell carcinoma (ESCC). However, its clinical significance and potential role in HCC remain unclear.

**Methods and results:**

In this study, expression of TUG1 was analyzed in 77 HCC tissues and matched normal tissues by using quantitative polymerase chain reaction (qPCR). TUG1 expression was up-regulated in HCC tissues and the higher expression of TUG1 was significantly correlated with tumor size and Barcelona Clinic Liver Cancer (BCLC) stage. Moreover, silencing of TUG1 expression inhibited HCC cell proliferation, colony formation, tumorigenicity and induced apoptosis in HCC cell lines. We also found that TUG1 overexpression was induced by nuclear transcription factor SP1 and TUG1 could epigeneticly repress Kruppel-like factor 2 (KLF2) transcription in HCC cells by binding with PRC2 and recruiting it to KLF2 promoter region.

**Conclusion:**

Our results suggest that lncRNA TUG1, as a growth regulator, may serve as a new diagnostic biomarker and therapy target for HCC.

**Electronic supplementary material:**

The online version of this article (doi:10.1186/s12943-015-0431-0) contains supplementary material, which is available to authorized users.

## Background

Hepatocellular carcinoma (HCC) is the dominant histological type of primary liver cancer which accounts for 70–85 % of primary malignancies in liver, and HCC is the third leading cause of cancer-related death worldwide [1]. While, half of these cases and deaths were estimated to occur in China [2]. Generally, hepatocarcinogenesis is a multistep process involving a number of genetic or epigenetic alterations that eventually result in the malignant transformation of hepatocytes [3, 4]. There have been significant advances in diagnosis and management of HCC and lots of therapeutic strategies have been improved [5]. However, the 5-year overall survival rate remains very poor and the biology of HCC remains poorly understood. Therefore, the identification of the new biomarkers for HCC and a detailed understanding of the molecular mechanisms underlying hepatic carcinogenesis will supply an arm for improving diagnosis and management of human HCC.

MicroRNAs and long noncoding RNAs (lncRNAs) are two major members of ncRNA family, and lots of studies have demonstrated that miRNAs play critical roles in HCC development [6]. For example, miR-331-3p could promote HCC cells proliferation and EMT-mediated metastasis by suppressiing PHLPP-mediated dephosphorylation of AKT [7]. LncRNAs, which are defined as being longer than 200 nucleotides without or with limit protein coding ability [8–10], emerge as essential regulators in almost all aspects of biology via regulation at chromatin organization, transcriptional and post-transcriptional levels [11, 12]. Additionally, a number of studies demonstrate that lncRNAs play an important role in tumorigenesis, and their misexpression confers tumor initiation, cancer cells growth and metastasis [13–15]. For example, lncRNA GAPLINC regulates CD44-dependent cell invasiveness by acting as a molecular decoy for miR211-3p and associates with poor prognosis in gastric cancer [16]. Moreover, lncRNA-ATB activated by TGF-β could promote the invasion -metastasis cascade in HCC cells by binding IL-11 mRNA, autocrine induction of IL-11 and triggering STAT3 signaling [17]. Although, there has been a heavy focus on the ways that lncRNAs contribute to cancers development, but their aberrant expression and functional roles in HCC development and diagnosis are still not well documented.

LncRNA TUG1,a 7.1-kb lncRNA, was firstly detected in a genomic screen for genes up-regulated in response to taurine treatment of developing mouse retinal cells [18]. Recently, TUG1 was found to be generally downregulated in NSCLC [19]. On the contrary,some studies showed that TUG1 can promote the cell proliferation of ESCC [20], urothelial carcinoma of the bladder [21] and osteosarcoma [22]. However, the functional role and underlying mechanism of TUG1 in HCC remains unclear. Here we investigated the relationship between TUG1 and HCC. We found that TUG1 was up-regulated in HCC tissues than that in corresponding non-tumor tissues and was related to tumor size and BCLC stage. Moreover, we found that TUG1 overexpression was induced by nuclear transcription factor SP1 and TUG1 could regulate cell growth both in vitro and in vivo via epigenetically silencing KLF2 by binding to PRC2. Our results suggest that TUG1 overexpression was induced by nuclear transcription factor SP1 and TUG1 can regulate KLF2 expression in the epigenetic level and facilitate the development of lncRNA-directed diagnostics and therapeutics of HCC.

## Results

### TUG1 is up-regulated in hepatocellular carcinoma tissues and is associated with tumor size and BCLC stage

**Fig. 1:**
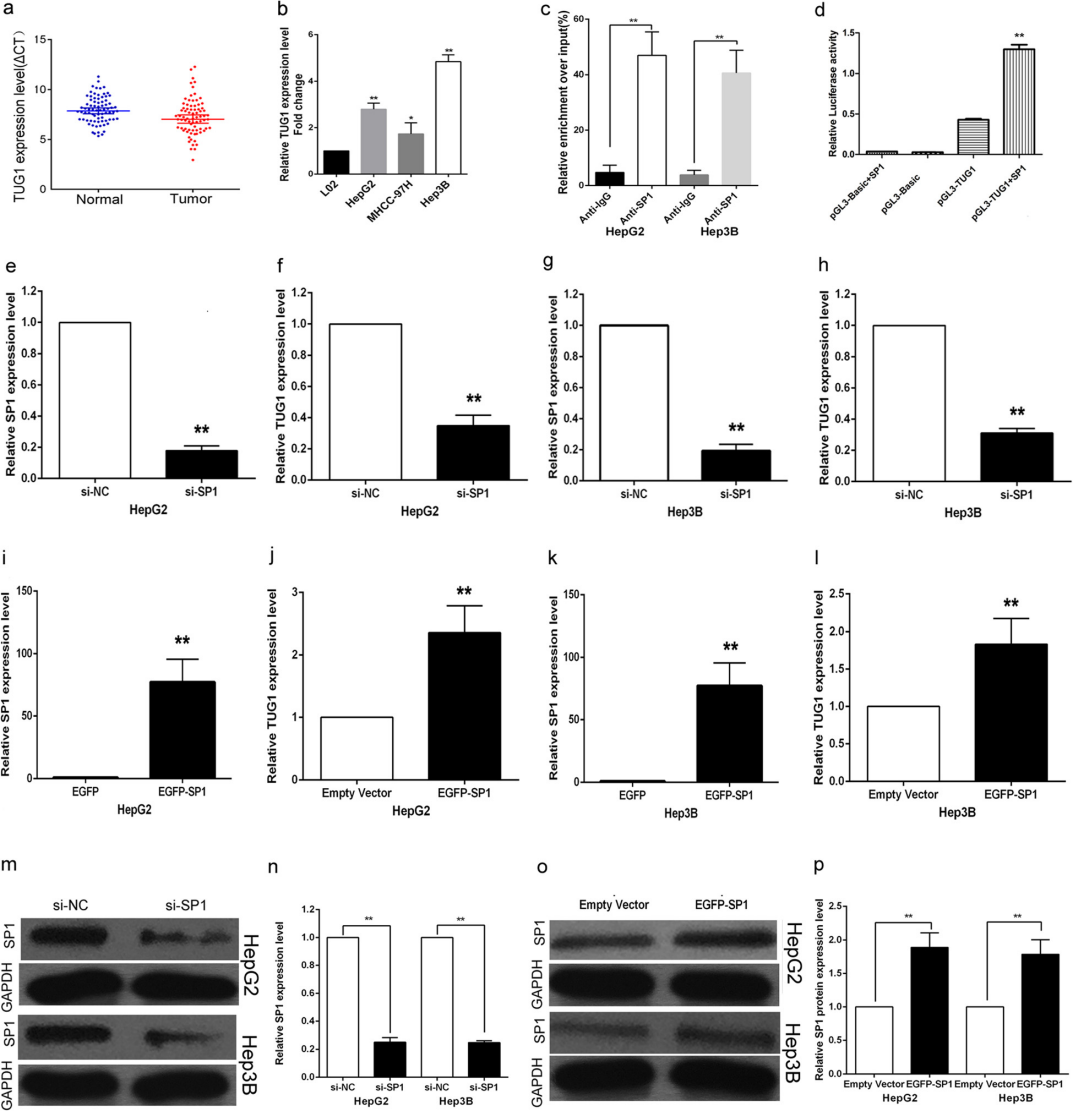
Relative TUG1 expression in HCC tissues and HCC cell lines and TUG1 was regulated by SP1. **a** Relative TUG1 expression in HCC tissues (*n* = 77) compared with corresponding non-tumor tissues (*n* = 77). TUG1 expression was examined by qPCR and normalized to GAPDH expression. Results were presented as ΔCT in tumor tissues relative to normal tissues. **b** Relative TUG1 expression levels of HCC cell lines (HepG2, Hep3B, MHCC-97H) compared with that in the normal hepatic epithelium cell line (L02). **c** ChIP–qPCR of SP1 occupancy and binding in the TUG1 promoter in HepG2 and Hep3B cells, and IgG as a negative control. **d** Luciferase reporter assay was performed by co-transfecting the TUG1 promoter fragment (pGL3-TUG1) or deleted TUG1 promoter fragment with SP1 expression vector or an empty vector in HepG2 cells. **e** The SP1 expression level was determined by qPCR when HepG2 cells transfected with si-SP1. **f** The TUG1 expression level was determined by qPCR when HepG2 cells transfected with si-SP1. **g** The SP1 expression level was determined by qPCR when Hep3B cells transfected with si-SP1. **h** The TUG1 expression level was determined by qPCR when Hep3B cells transfected with si-SP1. **i** The SP1 expression level was determined by qPCR when HepG2 cells transfected with EGFP-SP1. **j** The TUG1 expression level was determined by qPCR when HepG2 cells transfected with EGFP-SP1. **k** The SP1 expression level was determined by qPCR when Hep3B cells transfected with EGFP-SP1. **l** The TUG1 expression level was determined by qPCR when Hep3B cells transfected with EGFP-SP1. **m**,**n** The SP1 protein expression level was determined by Western blotting when HepG2/Hep3B cells transfected with si-SP1. **o**,**p** The SP1 protein expression level was determined by Western blotting when HepG2/Hep3B cells transfected with EGFP-SP1

The level of TUG1 was detected in 77 paired HCC tissues and corresponding adjacent normal tissues by qPCR, and normalized to GAPDH. The results showed that TUG1 expression was significantly up-regulated in 61.04 % (47 of 77,fold≧1.5) cancerous tissues compared with normal counterparts (*P* < 0.01) (Fig. 1a). To understand the significance of TUG1 overexpression in HCC, we investigated the potential associations between TUG1 expression and patients’ clinicopathological features. Clinicopathological features of HCC patients were shown in Table 1. Noticeably, high TUG1 expression was significantly correlated with tumor size (*P* = 0.003) and advanced BCLC stage (*P* < 0.01). However, TUG1 expression was not associated with other parameters such as drinking state (*P* = 0.531), age (*P* = 0.970), gender (*p* = 0.832), AFP (*P* = 0.570), HBV (*P* = 0.533) and cirrosis (*P* = 0.378) in HCC.

**Table 1:** Correlation between TUG1 expression and clinicopathological characteristics of HCC patients

Characteristics	TUG1		*P*-value
	High cases (No 47)	Low cases (No 30)	
Age (years)			0.970
< 50	19	12	
> 50	28	18	
Gender			0.832
Male	37	23	
Female	10	7	
Drinking state			0.531
Yes	28	20	
No	19	10	
HBV			0.533
Yes	40	27	
No	7	3	
Cirrosis			0.378
Yes	37	26	
No	10	4	
AFP			0.570
≦20	15	11	
20–400	14	11	
≧400	18	8	
Tumor size			0.003
≦3 cm	7	13	
3–5 cm	11	11	
5–10 cm	26	6	
≧10 cm	3	0	
BCLC stage			<0.01
0	1	3	
A	13	21	
B	33	6	

### TUG1 is up-regulated in HCC cell lines and could be activiated by transcript factor SP1

To investigate the functional role of TUG1 in HCC cells, qPCR was used to detect the expression of TUG1 in three HCC cell lines. As shown in Fig. 1b, HCC cell lines expressed higher levels of TUG1 compared with the normal hepatic epithelium cell line (L02). And we chose HepG2 and Hep3B for next study. We performed bioinformatics analysis and found that there are five SP1 binding sites in the TUG1 promoter region, which suggest that SP1 could also regulate TUG1 transcription (as shown in Table 2). In addition, over-expression of SP1 in HCC cells could up-regulate TUG1 expression, while knockdown of SP1 in HCC cells could down-regulate TUG1 expression (as shown in Fig. 1d-1o). ChIP assay showed that SP1 could directly bind to TUG1 promoter regions (as shown in Fig. 1c). Luciferase assay also showed that SP1 could directly bind to TUG1 promoter regions.

**Table 2:** Putative SP1-binding sites in the TUG1 promoter by JASPAR

Model ID	Model name	Score	Relative score	Start	End	Strand	Predicted site sequence
MA0079.3	SP1	17.396	1.000002277	1395	1405	1	GCCCCGCCCCC
MA0079.3	SP1	12.216	0.934831947	1571	1581	1	GTCCCTCCCCG
MA0079.3	SP1	14.434	0.962736926	1888	1898	1	CTCCCGCCCCC
MA0079.3	SP1	11.184	0.921848205	1894	1904	1	CCCCCGCCCTG
MA0079.3	SP1	14.626	0.965152506	1965	1975	1	GTCCCGCCCCT

### Knockdown of TUG1 inhibits HCC cell proliferation and induces cell apoptosis in vitro

**Fig. 2:**
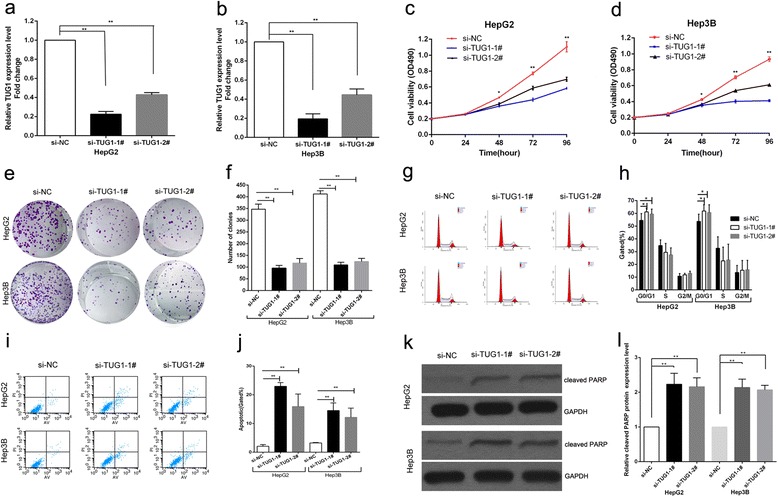
Effects of knockdown of TUG1 on HCC cells viability and apoptosis in vitro. **a**,**b** The TUG1 expression level was determined by qPCR when HepG2 and Hep3B cells transfected with si-TUG1. **c**,**d** MTT assays were used to determine the cell viability for si-TUG1-transfected HepG2 and Hep3B cells. Values represented the mean ± s.d. from three independent experiments. **e**,**f** Colony-forming assays were conducted to determine the proliferation of si-TUG1-transfected HepG2 and Hep3B cells. **g**,**h** Flow cytometry assays were performed to analysize the cell cycle progression when HCC cells transfected with si-TUG1 24 h later. The bar chart represented the percentage of cells in G0/G1, S, or G2/M phase, as indicated. **i**,**j** Flow cytometry assays were performed to analysis the cell apoptosis when HCC cells transfected with si-TUG1 48 h later. **k**,**l** PARP cleavage protein via western blot after TUG1 depletion. *P < 0.05, **P < 0.01

To investigate the potential role of TUG1 on HCC cells proliferation, TUG1 siRNA was transfected into HepG2 and Hep3B cells. To ensure the efficiency of interference and avoid off-target effects, we used two validated effective interference target sequence of TUG1, according to Zhang’s study [19]. QPCR assays revealed that TUG1 expression was significantly reduced after transfection with si-TUG1-1^#^ and si-TUG1-2^#^ (Fig. 2a). Then MTT assay showed that knockdown of TUG1 expression significantly inhibited cell proliferation both in HepG2 and Hep3B cells compared with control cells (Fig. 2b). Similarly, the result of colony-formation assay revealed that clonogenic survival was significantly decreased following inhibition of TUG1 in HepG2 and Hep3B cell lines (Fig. 2c). Next, flow cytometric analysis was performed to further examine whether the effect of TUG1 on proliferation of HCC cells by altering cell-cycle progression or apoptosis. The results revealed that the cell-cycle progression of HepG2/si-TUG1 and Hep3B/si-TUG1 was significantly stalled at the G1–G0 phase compared with cells transfected with si-NC (Fig. 2d). In addition, knockdown of TUG1 could obviously induce cell apoptosis (Fig. 2e).

### TUG1 promotes HCC cell proliferation in vivo

**Fig. 3:**
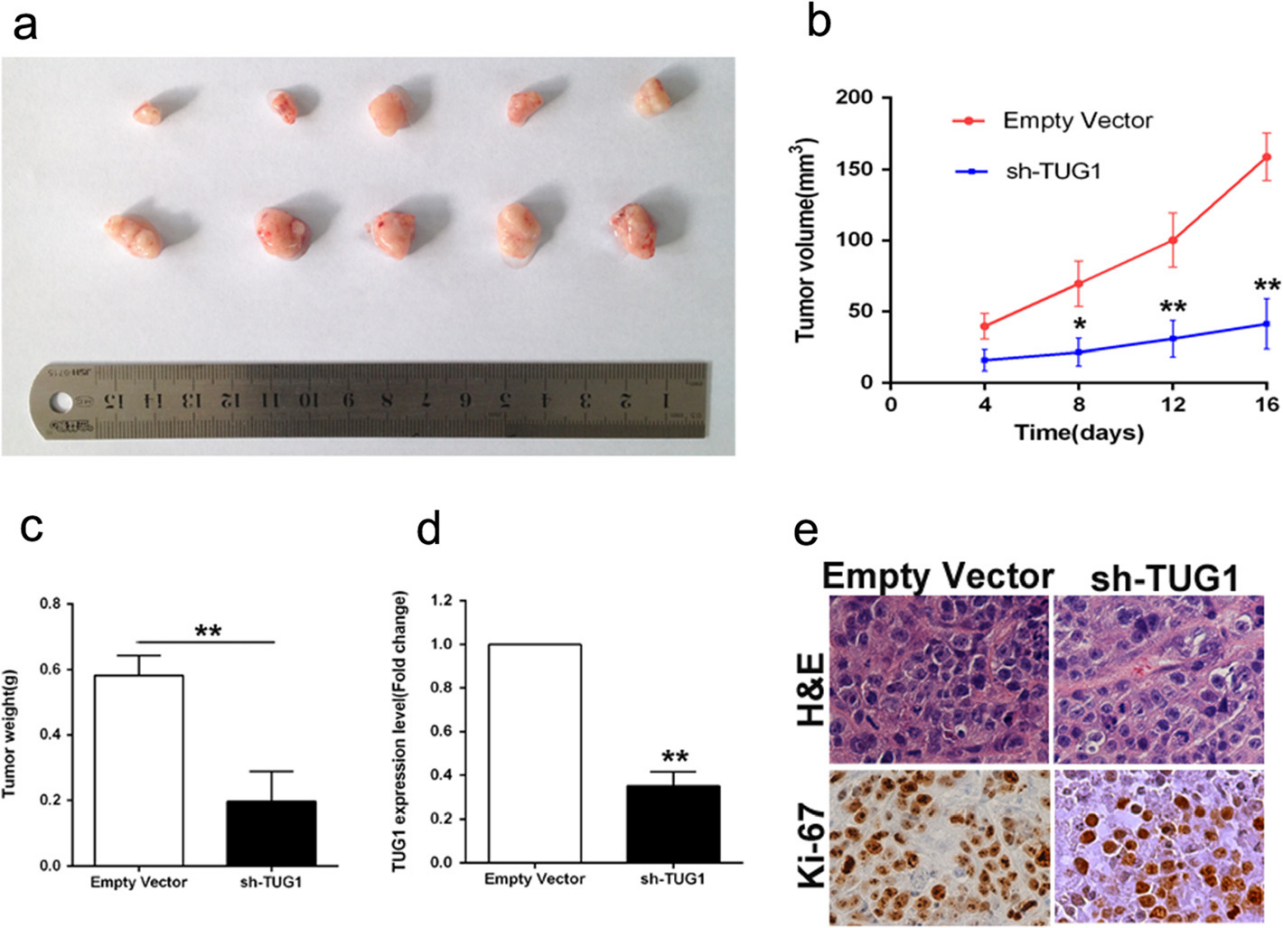
Effects of down-regulation of TUG1 on tumor growth in vivo. **a** Tumors from mice 16 days after injection of HepG2 cells stably transfected with sh-TUG1 or empty vector. **b** The tumor volume was calculated every four days after injection of HepG2 cells stably transfected with sh-TUG1 or empty vector. Points, mean (*n* = 5); bars indicate S.D. **c** Tumor weights are represented as means of tumor weights ± s.d. **d** QPCR analysis of TUG1 expression in tumor tissues formed from HepG2/sh-TUG1, HepG2/empty vector. **e** Tumors developed from sh-TUG1 transfected HepG2 cells showed lower Ki-67 protein levels than tumors developed by control cells. Left: H & E staining; Right: immunostaining. *P < 0.05, **P < 0.01

To further determine whether TUG1 affects tumorigenesis, we injected HepG2 cells transfected with either empty vector or sh-TUG1 into male nude mice. In consistent with in vitro results, tumor growth in sh-TUG1 group was obviously slower than that in the empty vector group (Fig. 3a). Up to 16 days after injection, the average tumor weight in sh-TUG1 group (0.196 ± 0.092 g) was significantly lower than that in the control group (0.582 ± 0.060 g) (*P* < 0.01) (Fig. 3b). qPCR analysis was performed to detect the average expression of TUG1 in tumor tissues selected from mice (Fig. 3c). Results demonstrated that the average expression level of TUG1 in sh-TUG1 group was lower than that in empty group (Fig. 3d). Moreover, we found that the tumors developed from empty vector transfected cells showed a stronger Ki-67 expression than that in tumors formed from sh-TUG1 as detected by IHC analysis (Fig. 3e). These data further supported the role of TUG1 in HCC cell growth and proliferation.

### TUG1 negatively regulates expression of KLF2

**Fig. 4:**
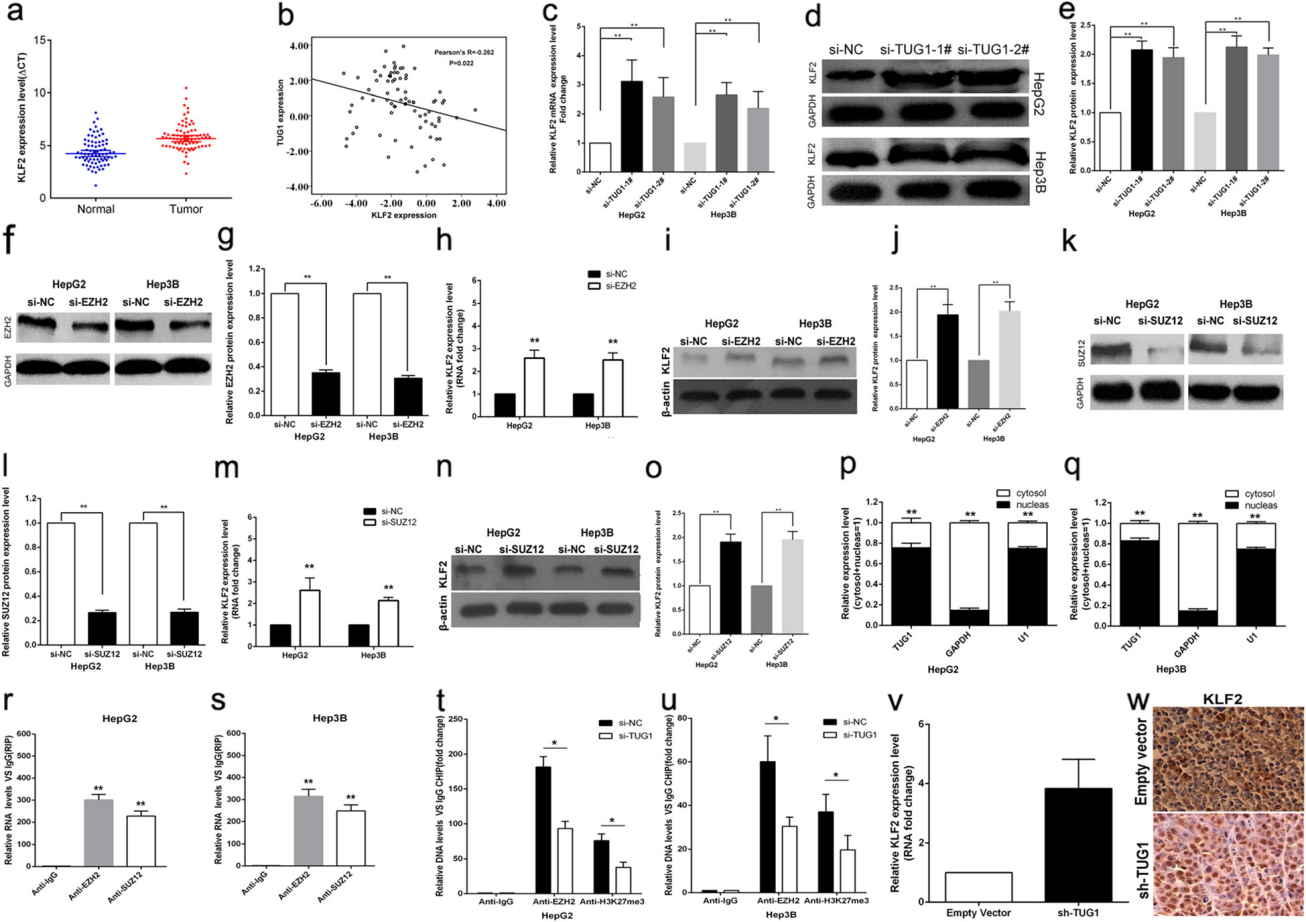
TUG1 could silence KLF2 expression. **a** KLF2 gene expression in HCC tissues (*n* = 77) compared with corresponding non-tumor tissues (*n* = 77). KLF2 expression was examined by qPCR and normalized to GAPDH expression. Results were presented as ΔCT in tumor tissues relative to normal tissues. **b** Co-expression analysis between TUG1 and KLF2. **c** The levels of KLF2 mRNA were detected by qPCR when HepG2 and Hep3B cells transfected with si-TUG1 and results are expressed relative to the corresponding values for control cells. **d**,**e** The levels of KLF2 protein levels were determined by Western Blotting when HepG2 cells transfected with si-TUG1. **f**,**g** The levels of EZH2 protein were detected by Western Blotting when HepG2 and Hep3B cells transfected with si-EZH2 and results are expressed relative to the corresponding values for control cells. **h** The levels of KLF2 mRNA were detected by qPCR when HepG2 and Hep3B cells transfected with si-EZH2 and results are expressed relative to the corresponding values for control cells. **i**,**j** The levels of EZH2 protein were detected by Western Blotting when HepG2 and Hep3B cells transfected with si-EZH2 and results are expressed relative to the corresponding values for control cells. **k**,**l** The levels of SUZ12 protein levels were determined by Western Blotting when HepG2 cells transfected with si-SUZ12. **m** The levels of KLF2 mRNA were detected by qPCR when HepG2 and Hep3B cells transfected with si-SUZ12 and results are expressed relative to the corresponding values for control cells. **n**,**o** The levels of KLF2 protein levels were determined by Western Blotting when HepG2 cells transfected with si-SUZ12. **p**,**q** TUG1 expression levels in cell cytoplasm or nucleus of HCC cell lines Hep3B and HepG2 were detected by qPCR. **r**,**s** RIP with rabbit monoclonal anti-EZH2, anti-SUZ12, anti-SNRNP70 and preimmune IgG from HepG2 and Hep3B cell extracts. RNA levels in immunoprecipitates were determined by qPCR. Expression levels of TUG1 RNA were presented as fold enrichment in EZH2 and SUZ12 relative to IgG immunoprecipitates; relative RNA levels of U1 snRNA in SNRNP70 relative to IgG immunoprecipitates were used as positive control. **t**,**u** ChIP–qPCR of EZH2 occupancy and H3K27-3me binding in the KLF2 promoter in HepG2 cells, and IgG as a negative control; ChIP–qPCR of EZH2 occupancy and H3K27-3me binding in the KLF2 promoter in HepG2 cells transfected with TUG1 siRNA (48 h) or scrambled siRNA. **v** The KLF2 expression level was determined by qPCR in mice tumors formed from HepG2/sh-TUG1,HepG2/empty vector. **w** Tumors developed from sh-TUG1 transfected HepG2 cells showed higher KLF2 protein levels than tumors developed by control cells. *P < 0.05, **P < 0.01 and N.S. not significant

As previously reported, TUG1 could regulate HOXAB7 expression by binding with PRC2. In the present study, we analysized the KLF2 gene expression in 77 paired HCC tissues and corresponding adjacent normal tissues by qPCR, and normalized to GAPDH. It showed that KLF2 was downregulated in HCC and negatively related to the expression of TUG1 by co-expression analysis (as shown in Fig. 4a,b). And we further found that knockdown of TUG1 expression could up-regulate both KLF2 mRNA and protein expression levels in HCC cells (Fig. 4c-e). Moreover, knockdown of EZH2 or SUZ12 could also up-regulate KLF2 mRNA and protein expression levels in HCC cells (Fig. 4f-o). We examined the TUG1 expression levels in HCC cell cytoplasm and nucleus distribution, and the results showed that TUG1 expression is more located in nucleus (seen in Fig. 4p,q). In addition, the results of RIP assays revealed that TUG1 could directly bind with PRC2 in HCC cells (seen in Fig. 4r,s). And ChIP assays were performed to determine whether EZH2 could directly bind to KLF2 promoter regions to silence KLF2 transcription. The results showed that EZH2 can directly bind to KLF2 promoter regions (616 bp), while knockdown of TUG1 expression decreased its’ binding ability (seen in Fig. 4t,u). Then qPCR analysis was performed to detect the average expression of KLF2 in tumor tissues selected from mice (Fig. 4v). Results demonstrated that the average expression levels of KLF2 in sh-TUG1 group was higher than that in either empty group. Finally, we found that the tumors developed from sh-TUG1 transfected cells showed a stronger KLF2 expression than that in tumors formed from empty vector as detected by IHC analysis (Fig. 4w). These data indicated that KLF2 is an new TUG1 target gene in HCC, and its’ expression can be silenced by EZH2 which is recruited by TUG1 to KLF2 promoter region and mediated H3K27 trimethylation modification.

### Over-expression of KLF2 impaires HCC cells proliferation and induces cell apoptosis

**Fig. 5:**
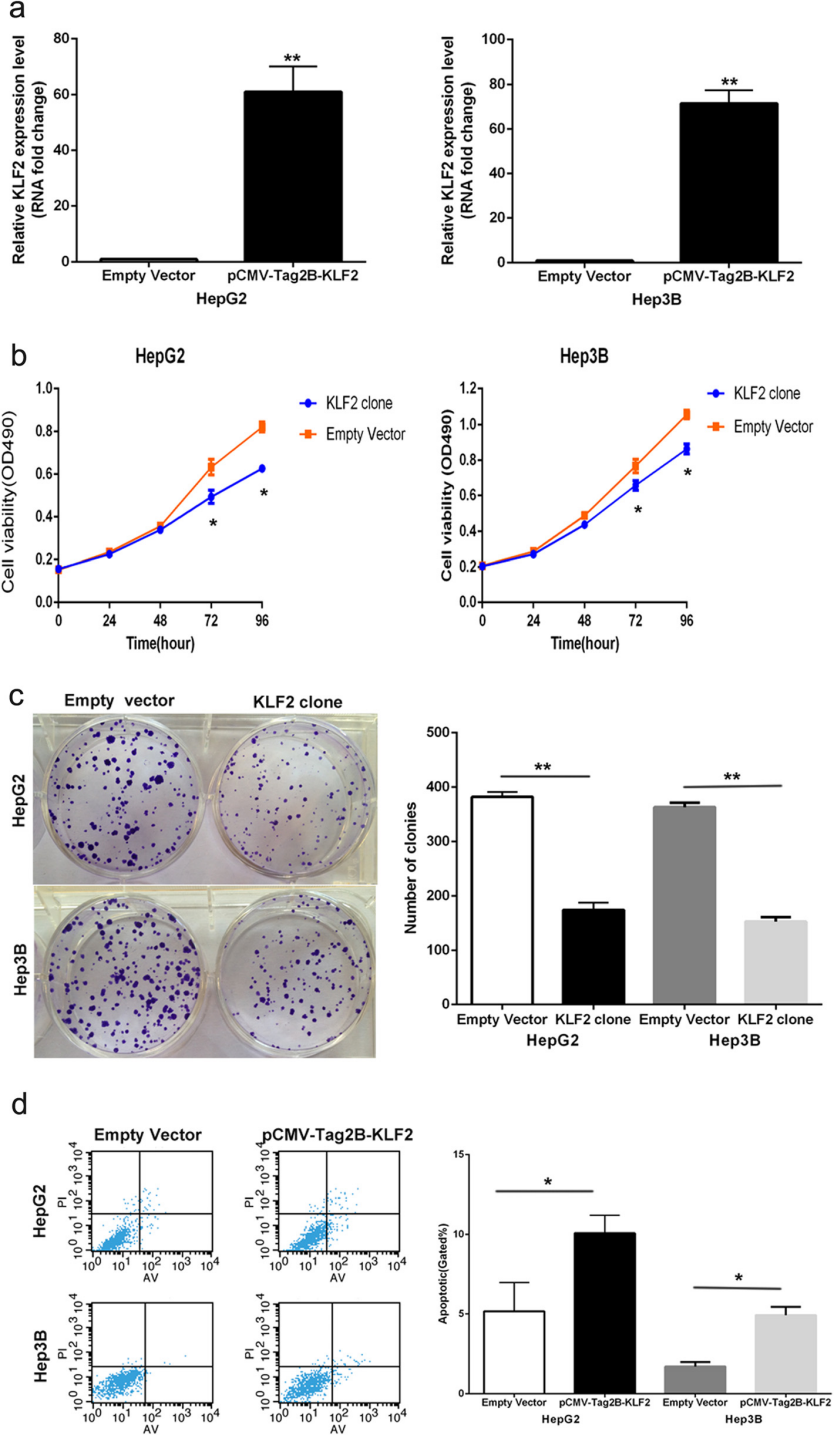
Over-expression of KLF2 expression inhibit HepG2 cells proliferation and improve apoptosis. **a** The mRNA level of KLF2 in HepG2 and Hep3B cells transfected with pCMV-Tag2B-KLF2 was detected by qPCR. **b**,**c** MTT assays and colony-forming assays were used to determine the cell viability for pCMV-Tag2B-KLF2 -transfected HepG2 and Hep3B cells. Values represent the mean ± s.d. from three independent experiments. **d** Apoptosis was determined by flow cytometry. UL, necrotic cells; UR, terminal apoptotic cells; LR, early apoptotic cells. *P < 0.05 and **P < 0.01

**Fig. 6:**
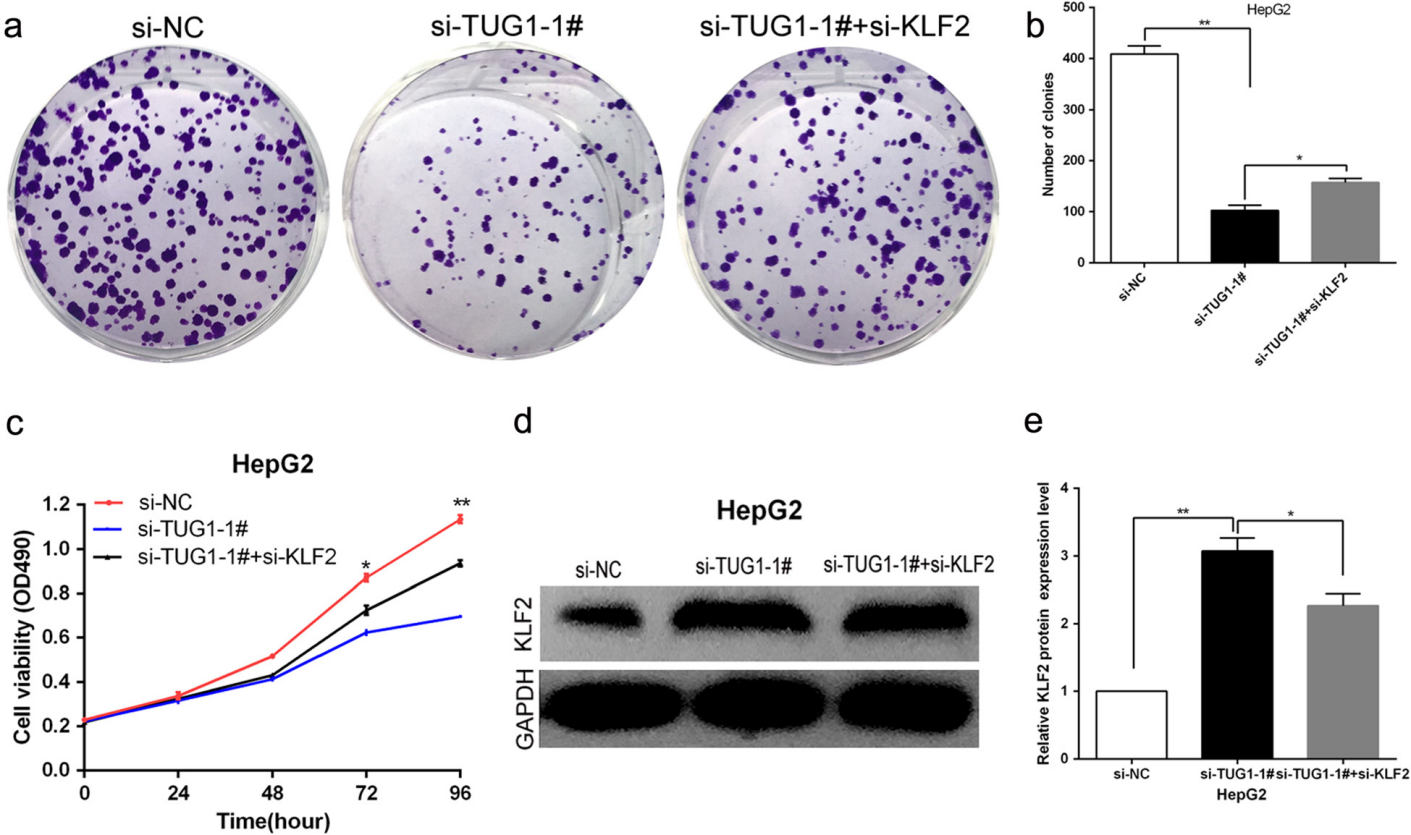
TUG1 negatively regulates expression of KLF2 by rescue assays. **a**,**b** Colony-forming assays were used to determine the cell viability for HepG2 cells transfected with si-NC,si-TUG1-1# and co-transfected with si-TUG1-1# and si-KLF2. Values represent the mean ± s.d. from three independent experiments. **c** MTT assays were used to determine the cell viability for HepG2 cells transfected with si-NC,si-TUG1-1# and co-transfected with si-TUG1-1# and si-KLF2. Values represent the mean ± s.d. from three independent experiments. **d**,**e** The levels of KLF2 protein levels were determined by Western Blotting when HepG2 cells transfected with si-NC, si-TUG1-1# and co-transfected with si-TUG1-1# and si-KLF2

To determine whether KLF2 involved in TUG1 mediated increased HCC cells proliferation, we up-regulated KLF2 expression in HCC cells by transfecting with a FLAG-tagged KLF2 expression vector using the pCMV-Tag2B vector (Stratagene, Santa Clara, CA, USA). The qPCR results showed that KLF2 expression is significantly up-regulated in pCMV-Tag2B-KLF2 transfected HCC cells when compared with control cells (Fig. 5a). Furthermore, MTT assays revealed that KLF2 over-expression inhibited HCC cells growth, and flow cytometric analysis indicated that increased KLF2 expression resulted in HCC cells G0/G1 arrest and induced cell apoptosis (Fig. 5b-d). These datas suggest that KLF2 partly involved in HCC cells proliferation and apoptosis. Moreover, to determine whether TUG1 regulate HCC cell proliferation by repressing KLF2 expression, rescue assays were performed. HepG2 cells were co-transfected with si-TUG1 and si-KLF2, and which was shown to rescue the decreased expression of TUG1 induced by knockdown of KLF2 (Fig. 6d, e). The results of MTT and colony formation assay results indicated that co-transfection could partially rescue si-TUG1-impaired proliferation in HepG2 cells (Fig. 6a, b, c). These data indicate that TUG1 promotes HCC cell proliferation through the down-regulation of KLF2 expression.

## Discussion

In the past decade, the discovery of numerous lncRNAs has dramatically altered our understanding of the biology of complex diseases including cancers. Recently, lots of studies have revealed that dysregulated expression of lncRNAs in multiple cancers, which may pinpoint the spectrum of cancer progression and predict patients’ outcome [23, 24]. For example, *FAL1* has been identified as an oncogenic lncRNA that associates with BMI1 and represses p21 expression in cancer by a functional genomic approach [25]. In HCC, HULC was the first reported lncRNA that is specifically up-regulated [26]. A number of lncRNAs, such as HULC [27] and LINC00974 [28] have been reported to be involved in HCC development and progression. In this study, we found that lncRNA TUG1 whose expression is significantly up-regulated in HCC tissues compared with normal tissues. Moreover, increased TUG1 expression was correlated with HCC tumor size and BCLC stage, which suggests that TUG1 may play a key role in HCC development and progression.

Several recent studies indicated that lncRNA expression could also be regulated by some transcript factors (TF), such as lincRNA-p21 expression can be regulated by p53 [29] and TINCR by SP1 [30]. TUG1 expression has been reported to be regulated by an key p53 [19]; however, we found that TUG1 expression could also be regulated by another TF SP1 in HCC cells, which suggests that one lncRNA may be simultaneously regulated by multiple different transcript factors. As is known, lncRNAs involved in cancer cells biological function, and we found that knockdown of TUG1 could impair HCC cells proliferation, invasion and induce cell apoptosis both in vitro and vivo. These data suggests that lncRNA TUG1 contributes to HCC development via regulation of cell proliferation and apoptosis.

TUG1 has been reported to regulate the expression of HOXB7 in NSCLC [19]. However, we found that TUG1 could bind with both EZH2 and SUZ12 in HCC cells. Furthermore, co-expression analysis indicated that KLF2 could be a new TUG1 downstream target, and knockdown of TUG1, EZH2 and SUZ12 expression indeed both up-regulated KLF2 expression levels in HCC cells. In addition, ChIP assays also demonstrated that EZH2 could directly bind to KLF2 promoter region and inhibition of TUG1 decreased its binding ability. Our results indicated that TUG1 could repress KLF2 transcription by binding with EZH2 and SUZ12 and recruitment of PRC2 to the KLF2 gene locus in HCC cells.

The Kruppel-like factor (KLF) family transcription factors have been identified as suppressors or activators of different genes in a cell type and promoter-dependent manner [31, 32]. Recently, lines of evidence showed that KLF members are emerging as tumor suppressors due to their roles in the inhibition of proliferation, invasion and induction of apoptosis [33]. As an member of KLF family, KLF2 expression is inactivated or lost in several cancers and possesses tumor-suppressor features mediated by KRAS [34]. Moreover, there is evidence showed that EZH2 could directly bind to KLF2 promoter and silence of KLF2 expression result in blocking the tumor-suppressor features of KLF2, which is partly mediated by p21 [35]. Our data also showed that TUG1 could take part in HCC cells proliferation by silencing KLF2 transcription, and KLF2 over-expression further led to the decreased HCC cells proliferation and increased cell apoptosis. Our results suggested that lncRNA, especially TUG1, may influence the same cell biological function via regulating different target genes depending on different cancer cells.

## Conclusion

To date, the possible targets and mechanism that underlie lncRNAs mediated regulatory behaviors still remain to be fully investigated in different cancers. In summary, the expression of TUG1 was significantly up-regulated in HCC tissues and cells, suggesting that its overexpression may be an important factor for HCC progression. We showed that TUG1 may regulates the proliferation ability of HCC cells partially through sliencing of the KLF2 by binding with PRC2, which suggested that lncRNAs contribute to different cancer cells biological function through regulating different genes. Our findings further the understanding of HCC pathogenesis, and facilitate the development of lncRNA-directed diagnostics and therapeutics against this disease.

## Materials and methods

### Patient data and tissue samples

A total of 77 fresh HCC tissue samples and matched normal adjacent tissue samples were selected from patients who underwent resection of primary HCC at Huai’an First People’s Hospital, Nanjing Medical University (Huai’an, China). The HCC diagnosis was histopathologically confirmed. None of the patients received preoperative therapy. Data from all subjects were obtained from medical records, pathology reports, and personal interviews with the subjects. The collected data included age, gender, drinking state, the history of HBV and cirrosis and HCC features (e.g., tumor size, stage). HCC clinical stage was determined according to the BCLC staging classification based on the article by Bruix and Llovet [36]. The clinical information for all of the samples is detailed in Table 1. Fresh samples were snap-frozen in liquid nitrogen immediately after resection and stored at −80 °C. Matched nontumor specimens were obtained from a part of the resected specimen that was farthest from the cancer.

### Ethical approval of the study protocol

This study was conducted according to the principles expressed in the Declaration of Helsinki. Tissue specimen collections were made with full informed consent of the patients, and followed institutional ethical guidelines that were reviewed and approved by Huai’an First People’s Hospital, Nanjing Medical University (Huai’an, China).

### Cell culture

Human HCC cell lines (HepG2, MHCC-97H, Hep3B) and one normal hepatic epithelial cell line (L02, control) were provided by Dr Beicheng Sun from the Department of Hepatopancreatobiliary, First Affiliated Hospital, Nanjing Medical University (Nanjing City, Jiangsu Province, P. R. China). All cell lines were cultured in DMEM (GIBCO-BRL) medium supplemented with 10 % fetal bovine serum (FBS) at 37 °C in 5 % CO2.

### RNA extraction and qPCR analysis

The total RNA was extracted from tissues or cultured cells with TRIzol reagent (Invitrogen, Grand Island, NY, USA), according to the manufacturer’s protocol. One microgram total RNA was reverse transcribed in a final volume of 20 μL under standard conditions using PrimeScript RT Reagent Kit with gDNA Eraser (Takara, Dalian, China; RR047A). After the RT reaction, 1 μL of the complementary DNA was used for subsequent qPCR reactions (SYBR Premix Ex Taq, TaKaRa) following the manufacturer’s protocol. The results were normalized to the expression of GAPDH. The qPCR and data collection were carried out on ABI 7500 real-time PCR system (Applied Biosystems, Foster City, CA, USA), and results were analyzed and expressed relative to threshold cycle(CT) values, and then converted to fold changes. All primer sequences are summarized in Additional file 1: Table S1.

### Transfection of cell lines

HCC cell lines were transfected with specific siRNA oligonucleotidesby using Lipofectamine RNAi MAX, according to the manufacturer’s protocol (Invitrogen). TUG1 siRNA, to avoid off-target effects and ensure the efficiency of interference, we used two indeed effective interference target sequence of TUG1, according to previous study [19]. EZH2 siRNA was purchased from Realgene (Nanjing, China). Non-specific siRNA (si-NC) was purchased from Invitrogen. Typically, cells were seeded at six-well plates and then transfected the next day with specific siRNA (100 nM) and control siRNA (100 nM). EGFP-SP1 was purchased from Add gene. Plasmid vectors (EGFP-SP1, sh-TUG1 pCMV-Tag2B-FLAG-KLF2 and empty vector) for transfection were prepared using DNA Midiprep or Midiprep kits (Qiagen, Hilden, Germany), and transfected into HepG2 and Hep3B cells.

### Cell proliferation assays

Cell proliferation was monitored by Cell Proliferation Reagent Kit I (MTT) (Roche, Basel, Switzerland). The transfected cells were plated in 96-well plates (3000 cells/well). Cell proliferation was determined every 24 h following the manufacturer’s protocol. For the colony-formation assay, 500 transfected cells were placed into each well of a six-well plate and maintained in DMEM containing 12 % FBS for 12 days, replacing the medium every 4 days. Colonies were fixed with methanol and stained with 0.1 % crystal violet (Sigma-Aldrich, St. Louis, MO, USA) in PBS for 15 min. The colony formation was determined by counting the number of stained colonies. Triplicate wells were measured in each treatment group.

### Flow cytometry for cell cycle analysis

HepG2 or Hep3B cells for cell cycle analysis were collected 24 h after transfected with si-TUG1 or respective control, 48 h after transfected with pCMV-Tag2B-KLF2 or empty vector. Then cells were stained with PI using the CycleTEST™ PLUS DNA Reagent Kit (BD Biosciences) according to the protocol and analyzed by FACScan. The percentage of the cells in G0/G1, S, and G2/M phase were counted and compared.

### Flow cytometry for cell apoptosis analysis

HepG2 or Hep3B cells transfected with si-TUG1, pCMV-Tag2B-KLF2 or respective control were harvested 48 h and then collected. After the double staining with FITC-Annexin V and Propidium iodide (PI) was done using the FITC Annexin V Apoptosis Detection Kit (BD, Biosciences) according to the manufacturer’s protocol, the cells were analyzed with a flow cytometry (FACScan®; BD Biosciences) equipped with a CellQuest software (BD Biosciences). Cells were discriminated into viable cells, dead cells, early apoptotic cells, and apoptotic cells, and then the relative ratio of early apoptotic cells were compared to control transfectant from each experiment.

### Cell migration and invasion assays

HepG2 or Hep3B cells transfected with si-TUG1or respective control were harvested 48 h and then collected. For the migration assays, 5 × 10^4^ cells in serum-free medium were placed into the upper chamber of an insert (8 μm pore size; Millipore). For the invasion assays, 1 × 10^5^ cells in serum-free medium were placed into the upper chamber of an insert coated with Matrigel (Sigma-Aldrich). Medium containing 10 % FBS was added to the lower chamber. After incubation for 24 h, we removed the cells remaining on the upper membrane with cotton wool. Cells that had migrated or invaded through the membrane were stained with methanol and 0.1 % crystal violet, imaged, and counted using an IX71 inverted microscope (Olympus, Tokyo, Japan). Experiments were repeated three times.

### Xenograft study

HepG2 cells were transfected with sh-TUG1 or Scramble using Lipofectamine 2000 (Invitrogen). After 48 h, cells were collected and injected into either side of the posterior flank of the male BALB/c nude mice (4–5 weeks old). Mice were purchased from Shanghai Experimental Animal Center of the Chinese Academy of Sciences. The tumor volumes and weights were measured every 4 days in mice from the control (5 mice) or sh-TUG1 (5 mice) groups, and tumor volumes were calculated by using the equation *V* = 0.5 × D × d2 (V, volume; D, longitudinal diameter; d, latitudinal diameter). Sixteen days after injection, the mice were killed and tumor weights were measured and used for further analysis. This study was carried out strictly in accordance with the recommendations in the Guide for the Care and Use of Laboratory Animals of the National Institutes of Health. The protocol was approved by the Committee on the Ethics of Animal Experiments of Nanjing Medical University.

### Immunohistochemistry

Tumors from mice were immunostained for HE, ki-67 and KLF2. The signal was amplified and visualized with 3′-diaminobenzidine chromogen, followed by counterstaining with hematoxylin. Expression was considered to be positive when 50 % or more tumor cells were stained. Anti-ki-67(1:50) and anti-KLF2(1:50) were purchased from R&D company.

### Western blot assay

The cells were lysed by using mammalian protein extraction reagent RIPA (Beyotime, Haimen, China) supplemented with protease inhibitors cocktail (Roche). Fifty micrograms of the protein extractions were separated by 10 % SDS-PAGE transferred to 0.22 mm nitrocellulose (NC) membranes (Sigma-Aldrich) and incubated with specific antibodies. The autoradiograms were quantified by densitometry (Quantity One software; Bio-Rad, Hercules, CA, USA). Anti-KLF2 was purchased from Sigma (1:1000). Results were normalized to the expression β-actin (Mouse anti-β-actin) (Sigma (1:1000)).

### Subcellular fractionation location

The separation of the nuclear and cytosolic fractions of HCC cell lines was performed according to the protocol of the PARIS Kit (Life Technologies, Carlsbad, CA, USA).

### Chromatin immunoprecipitation assays(ChIP)

The ChIP assays were performed by using EZ-ChIP KIT according to the manufacturer’s instruction (Millipore, Billerica, MA, USA). HepG2 or Hep3B cells were treated with formaldehyde and incubated for 10 min to generate DNA-protein cross-links. Cell lysates were then sonicated to generate chromatin fragments of 200–300 bp and immunoprecipitated with EZH2 and H3K27me3-specific antibody (CST) or IgG as control. Precipitated chromatin DNA was recovered and analyzed by qPCR.

### RNA immunoprecipitation(RIP)

RIP experiments were performed by using a Magna RIP RNA-Binding Protein Immunoprecipitation Kit (Millipore) according to the manufacturer’s instructions. Antibody for RIP assays of EZH2 and SUZ12 were purchased from Millipore.

### Statistical analysis

All statistical analyses were performed by using SPSS 17.0 software (IBM, Chicago, IL, USA). The significance of differences between groups was estimated by the Student *t*-test, Wilcoxon test or *χ*2 test. Two-sided p-values were calculated, and differences were considered to be statistically significant at *P* < 0.05. Kendall’s Tau-b and Pearson correlation analyses were used to investigate the correlation between TUG1 and KLF2 expressions.

## Additional file

Additional file 1: Table S1. Sequence of primers and siRNA. (XLS 11 kb)
